# Radiation-induced changes in salivary metabolite profile and pathways in head and neck cancer patients

**DOI:** 10.1007/s00784-025-06225-4

**Published:** 2025-02-21

**Authors:** Saga Ramsay, Eelis Hyvärinen, Wilfredo González-Arriagada, Tuula Salo, Marcio Ajudarte Lopes, Jopi J. W. Mikkonen, Bina Kashyap, Arja M. Kullaa

**Affiliations:** 1 Institute of Dentistry, School of Medicine, University of Eastern Finland, Kuopio Campus, Kuopio, 70210 Finland; 2 Educational Dental Clinic, Kuopio University Hospital, The Wellbeing Services County of North Savo, Kuopio, Finland; 3 Facultad de Odontología, Universidad de los Andes, Santiago, Chile; 4 Centro de Investigación E Innovación Biomédica, Universidad de los Andes, Santiago, Chile; 5 IMPACT-Center of Interventional Medicine for Precision and Advanced Cellular Therapy, Santiago, Chile; 6 Department of Pathology, University of Helsinki and Helsinki University Hospital, Helsinki, Finland; 7 Department of Oral and Maxillofacial Diseases, Faculty of Medicine, University of Helsinki, Helsinki University Hospital, ClinicumHelsinki, Finland; 8 Translational Immunology Research Program (TRIMM), University of Helsinki, Helsinki, Finland; 9 CAN Digital Precision Cancer Medicine Flagship, University of Helsinki, Helsinki, Finland; 10 Research Unit of Population Health, Faculty of Medicine, University of Oulu, Oulu, Finland; 11 Medical Research Center, Oulu University Hospital, University of Oulu, Oulu, Finland; 12 Department of Oral Diagnosis, School of Dentistry, State University of Campinas, Sao Paulo, CEP 13414-018 Brazil

**Keywords:** Radiotherapy, Saliva, Metabolites, Metabolomics, NMR spectroscopy, Head and neck cancer

## Abstract

**Introduction:**

This longitudinal study assessed the salivary metabolic profile in patients with head and neck cancer (HNC) treated with radiotherapy (RT). This study aims to investigate salivary metabolites and biological oral pathways induced by RT.

**Methods:**

Clinical data and unstimulated whole-mouth saliva (USWMS) were obtained from 45 HNC patients before, during, and one week after the RT. Data was also collected from 30 healthy controls. NMR spectroscopy identified and quantified 24 metabolites. Spearman’s rank correlation analysis and pathway enrichment analysis (MetaboAnalyst 6.0) was performed to check the effect of cancer therapy on the correlation and pathways of different salivary metabolites.

**Results:**

Of 24 metabolites identified, 17 salivary metabolites showed a consistent decrease in the concentration during and after treatment of HNC patients. The metabolite proline decreased, whereas fucose and 1,2-Propanediol were increased in the saliva causing altered redox balance and abnormal fucosylation in HNC patients compared to controls. Spearman correlation analysis indicated changes between pyruvate and some other metabolites, including alanine, trimethylamine, choline, taurine, and succinate, during RT. Five pathways (Pyruvate metabolism; Glycolysis / Gluconeogenesis; Glycine, serine, and threonine metabolism; Glyoxylate and dicarboxylate metabolism; and Alanine, aspartate and glutamate metabolism) are affected, demonstrating the metabolic dysregulation due to RT. The pyruvate metabolism was overpresented with the high Pathway Impact score.

**Conclusion:**

Salivary metabolomics analysis revealed significant alterations in the metabolic profile of HNC patients undergoing RT, providing valuable insights into treatment-induced oral pathobiological changes. Alterations in salivary pathways during RT suggest disturbances in redox homeostasis, oxidative stress, and inflammation. The ability to monitor salivary metabolites and pathways non-invasively holds promise to personalized medicine in HNC treatment by enabling early detection of treatment-related toxicities, monitoring treatment response, and tailoring interventions to patient needs.

**Supplementary Information:**

The online version contains supplementary material available at 10.1007/s00784-025-06225-4.

## Introduction

Head and neck cancer (HNC) remains a significant global health concern, with increasing mortality rates during the last decade, reflecting a rising incidence and stagnant survival rates. Among HNCs, the oral cavity accounts for over 389,485 new cases and 188,230 deaths annually [1]. While the conventional treatment methods are surgery, radiotherapy (RT), and chemotherapy for HNCs, RT is crucial for managing HNC, either as a primary treatment or adjuvant to surgery [2]. The radiation dose of ≥ 60 Grays (Gy) is administered in fractionated daily doses. Despite advancements in treatment, early diagnosis and effective management of RT-induced complications remain critical for improving patient outcomes.

The administered radiation causes a certain degree of biological damage depending on the radiation field, type, and dose [3]. RT can lead to various adverse effects, including changes in the salivary flow and composition, oral mucositis, taste dysfunction, trismus, changes in oral microbiome, the atrophy of bone, and soft tissue fibrosis. These complications not only impact patients’ quality of life but also increase their risk of infection and long-term oral health problems [4]. Therefore, identifying the early indicators of RT-induced complications is crucial for implementing timely interventions and improving patient care.

The saliva is an emerging diagnostic biofluid widely used for diagnostic purposes due to its accessibility and rich composition, including a diverse array of metabolites. Saliva has a complex composition and contains the end-products of metabolic pathways called metabolites [5]. Metabolites are sensitive to systemic changes and offer valuable insights into physiological and pathological processes related to the patient’s state [6]. In recent years, the metabolic profile of saliva by various analytic techniques such as high-resolution nuclear magnetic resonance (1H-NMR) spectroscopy, high-performance liquid chromatography-mass spectroscopy (HPLC–MS), two-dimensional gas chromatography (2DGC–MS), Fourier-transform infrared (FTIR) spectroscopy has discovered discriminatory metabolic signatures for several diseases [7]. Previous studies have been demonstrated the potential of NMR-based salivary metabolomics in differentiating HNC patients from healthy individuals [8–12] and in monitoring the treatment response in other oral diseases [13].

Oral microbes have been increasingly linked to cancer and salivary metabolites, and an emerging concept in cancer implies that oral microbes are able to modulate the carcinogenic process [14]. The salivary metabolome, influenced by oral microbes, can serve as a diagnostic tool for investigating the biological function of oral toxicities related to cancer therapy [15]. Oral dysbiosis, either directly or indirectly via immunosuppression, oxygen deprivation, biofilm formation, or other potential mechanisms, can cause the loss of beneficial oral species and subsequent pathogen colonization [16]. The alteration in salivary metabolites of cancer patients undergoing RT and identifying the metabolites before and after RT in HNC patients will help in understanding the oral microenvironment changes [17]. Dysbiosis has been considered as a hallmark of oral cancer, with certain bacteria proposed as potential biomarkers [18].

While some studies have explored alterations in salivary metabolites in HNC patients, research specifically investigating the impact of RT on the salivary metabolome remains limited [17, 18]. Determining salivary metabolites using NMR in patients undergoing RT can provide pathobiological information on radiation-induced changes in the salivary pathways. We hypothesized that salivary metabolites and their pathways could provide detailed information on biological oral changes in HNC patients undergoing RT. To test this hypothesis, we conducted a longitudinal study to assess the salivary metabolic profile of HNC patients before, during, and after RT. To test this hypothesis, we conducted a longitudinal study to assess the salivary metabolic profile of HNC patients before, during, and after RT.

## Materials & methods

### Ethics

The present study was approved by the Ethics Committee for Human Studies, Piracicaba Dental School, Brazil (protocol number: 142/2010) and by the Research Ethics Committee of the Northern Savo Hospital District (protocol number 754/2018; 28.11.2023). The present study fulfilled the World Medical Association Declaration (Helsinki, Finland, 1964). All patients included in the study signed an informed consent form for participation and the patient information was anonymous after sample collections.

### Subjects and clinical data

This clinical longitudinal study included 45 patients with HNC and 30 healthy controls. The HNC patients received RT in the Oncology centre. Of 45 patients, 22 HNC patients also received chemotherapy: cisplatin and 5-fluorouracil one day per week. RT was given 5 days a week with a dose of 1.8 to 2 Gy daily, with a total dose of 66 to 74 Gy. All the patients received conventional dental treatment before they were given RT. Patients underwent weekly check-ups during and after the treatments. The appearance of treatment side effects was evaluated during the weekly examinations. The presence of hyposalivation, dysgeusia, dermatitis and mucositis was recorded. All demographic details of HNC patients are presented in Table 1. The control group contains 19 male and 11 female non-smoking subjects with a mean age of 54.4 years (range 42–74 years) without the history of any cancer.

**Table 1:** Demographic and clinical characteristics of head and neck cancer (HNC) patients (*N* = 45) included in the study

Gender	
Male	38 (84.4%)
Female	7 (15.6%)
Age (years)	
Mean age	58
Range	40 – 85
Tumour location	
Hypopharynx	2 (4.4%)
Larynx	15 (33.3%)
Oral cavity	17 (37.8%)
Oropharynx	4 (8.9%)
Salivary gland	2 (4.4%)
Unknown primary	5 (11.1%)
Histological diagnosis	
SCC	43 (95.6%)
ACC	2 (4.4%)
Risk factors	
Smoking	
Male	35/38
Female	2/7
Drinking	
Male	33/38
Female	1/7
Total dose of RT (Gy)	
66—74	
Complications (Male / Female)	
Mucositis	64% (25/4)
Candidiasis	58% (24/2)
Xerostomia	80% (31/5)
Hyposalivation	56% (19/6)
Dysgeusia	78% (31/4)
Dermatitis	93% (36/6)

*SCC* Squamous cell carcinoma

*ACC* Adenoid cystic carcinoma

### Saliva collection

**Fig. 1:**
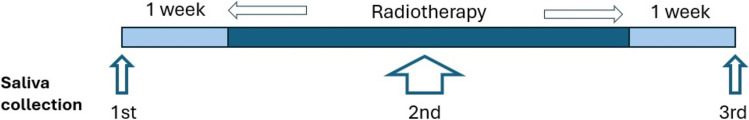
Workflow of saliva collection in HNC patients at three different time points; 1st one week before radiotherapy (RT), 2nd during RT, and 3rd one week after RT

Saliva samples were collected from the HNC patients one week before treatment and during treatment (third week of treatment), as well as one week after the completion of the RT treatment (Fig. 1). Samples were collected in the morning between 9 and 11 am to minimize the interference of the circadian rhythm. The patients abstained from drinking, eating, and brushing their teeth for at least one hour before the collection. Unstimulated whole mouth saliva was collected by allowing the patients to let naturally produced saliva drain into a sterile glass cup for five minutes. The flow rate (ml/min) was assessed immediately after the collection and was considered normal if it was 0.3 ml/min or higher. When the salivary flow rate is less than 0,3 ml/min it is considered as hyposalivation. The samples were immediately transferred to the laboratory where they were centrifuged at 14 000 rpm for 6 min at 18 °C to delete any solid unneeded parts (debris). The supernatants were stored immediately at -80 °C for NMR analysis.

### NMR analysis

After thawing at room temperature, 450 µl of each saliva sample was mixed with 50 µl of NMR-buffer (1.5 M KH2PO4, 2 mM NaN3, 5.8 mM sodium 3-(trimethylsilyl) propionate-2,2,3,3-d4, D2O, pH 7.4). After vortexing, the obtained mixture (500 µl) was then transferred to NMR tubes. The samples were stored at + 6 °C in the sample changer until the measurement.

All NMR data was acquired using a Bruker AVANCE III HD spectrometer operating at 600.2 MHz for the proton which was equipped with a highly selective cryoprobe that includes an automatic tuning and matching unit (Bruker CryoPrope Prodigy; Bruker BioSpin GmbH, Rheinstetten, Germany). The control of the spectrometer occurred by TopSpin 3.2 software (Bruker BioSpin GmbH). Topshim was used to shim each saliva sample. After that, samples were preheated to + 25 °C 30 min before the measurement. The NMR data were recorded (128 k data points) at temperature + 25 °C with a 5.8 s repetition time (relaxation time 3.0 s, acquisition time 2.8 s). Before recording, 4 dummy scans using 64 transients with an automatically calibrated 90° pulse to achieve the required signal-to-noise level. A Bruker cpmg1d pulse sequence with t2-filter time of 80 ms and irradiation field of 50 Hz was used to quieten the water peak. For each sample, the automatic calibration of the pulse 90° was used. For all the samples, a constant receiver gain was set.

### Data processing

The acquired NMR spectra were manually corrected for the phase using TopSpin 3.2 software (Bruker BioSpin GmbH). Before Fourier transformations to spectra, the measured free induction decays were multiplied with an exponential window function with a 1.0 Hz line broadening. The total-line-shape fitting tool in NMR software (PERCH Solutions Ltd, Kuopio, Finland) was used in the metabolite quantification. The PERCH software allows the accurate quantification of identified metabolites even if the signals are overlapping, or the baseline is not linear due to the heavy protein background envelope or overlapping signals [19]. An internal reference compound (tri-methylsilyl-propanoic acid, TSP) with known concentration, was used as an internal standard. Obtained final metabolite concentrations in saliva are described as µmol/l.

### Statistics and metabolic pathway analysis

The Shapiro–Wilk test and the kurtosis and skewness values were used to test the distribution of metabolic concentrations for normality. Since the metabolite concentrations were not normally distributed, non-parametric Mann–Whitney U-test was done to compare salivary metabolite concentrations between HNC patients and controls, as well as metabolites in HNC patients at different collection stages. The statistical significance was set at *p* < 0.05. Spearman’s rank correlation analysis was carried out to check the effect of cancer therapy on the correlation between different metabolites and their association with metabolic pathways. The strength of dependence (correlation) was classified as follows: < 0.00 no correlation; < 0.40 weak correlation; 0.40 – 0.60 moderate correlation; 0.60 – 0.80 strong correlation; > 0.80 very strong correlation. All statistical analyses were conducted using SPSS software, version 27.0 (IBM Corp., Armonk, NY, USA). Pathway enrichment analysis was performed using MetaboAnalyst 6.0 [20] (http://www.metaboanalyst.ca/).

## Results

The HNC patient demographic data including tumor characteristics, risk factors, histological diagnosis, and radiation induced complications are presented in Table 1.

A total of 24 salivary metabolites were detected and their concentrations were compared between HNC patients and healthy controls (Table 2). Statistical analysis revealed a lower median concentration of proline (*p* = 0.030**)** and a higher concentration of 1,2-propanediol (propylene glycol) and fucose (*p* = 0.032 and *p* < 0.001, respectively) in HNC patients compared with controls. No significant differences were observed for other metabolites between HNC patients and healthy controls.

**Table 2:** Comparison of salivary metabolite concentrations between patients with head and neck squamous cell carcinoma (HNC, *n* = 42) and healthy controls (*n* = 30)

Salivary metabolite	Before treatment Mean ± SD	Controls Mean ± SD	*p*-value
SCFAs			
Acetate	2916.1 ± 2582.8	1882.4 ± 1108.9	0.765
Butyrate	89.3 ± 41.0	58.6 ± 25.4	0.562
Formate	228.7 ± 51.7	179.2 ± 50.8	0.428
Propionate	677.3 ± 481.4	527.3 ± 381.0	0.562
Amino acids			
Alanine	92.3 ± 31.1	107.4 ± 23.2	0.820
Glycine	560.8 ± 84.9	499.3 ± 92.8	0.528
Histidine	23.3 ± 11.8	#	-
Phenylalanine	82.4 ± 22.5	75.7 ± 13.8	0.847
Taurine	132.4 ± 87.1	170.2 ± 32.0	0.562
Proline	182.8 ± 151.2	634.4 ± 185.0	0.030*
Tyrosine	115.8 ± 17.9	99.8 ± 14.4	0.847
Amines			
Trimethylamine	0.8 ± 0.6	1.9 ± 1.1	0.392
Methylamine	5.7 ± 1.2	3.3 ± 1.9	0.445
Organic acids			
Citrate	12.0 ± 8.9	26.7 ± 14.4	0.263
Lactate	207.5 ± 34.7	197.4 ± 27.4	0.986
Pyruvate	32.1 ± 29,0	17.9 ± 13.4	0.515
Succinate	59.9 ± 19.8	68.9 ± 15.1	0.765
Others			
Choline	21.5 ± 12.2	19.2 ± 7.2	0.765
Isopropanol	2.0 ± 1.2	2.6 ± 2.3	0.394
1,2-Propanediol	69.3 ± 19.8	29.1 ± 21.1	0.032*
Butanol	71.28 ± 52.7	56.5 ± 47.6	0.428
Ethanol	30.6 ± 21.6		
Fucose	694.0 ± 115.5	179.1 ± 87.8	< 0.001***
Methanol	119.3 ± 5.1	85.4 ± 8.3	0.445

*P*-values are based on Mann–Whitney U test. (# conentrations were below the detection limit)

Salivary metabolite analysis before, during, and after the treatment of HNC patients showed significant differences in the metabolite concentrations, and most of the metabolite concentrations are decreased after RT (Table 3). The concentrations of trimethylamine and citrate were slightly increased, and that of methylamine was significantly increased (*p* = 0.392; *p* = 0.263; *p* < 0.01 respectively). Histidine and ethanol were below the detection limit in the saliva samples of HNC patients after treatment.

**Table 3:** Salivary metabolites (µM) before, during, and after radio therapy in HNC patients

Salivary metabolite	Before treatment Mean ± SD	During treatment Mean ± SD	After treatment Mean ± SD	*p*-value
SCFAs				
Acetate	2916.1 ± 2582.8	2663.8 ± 2108.9	1503.0 ± 715.2	0.256
Butyrate	89.3 ± 41.0	104.8 ± 85.8	16.3 ± 10.9	0.001***
Formate	228.7 ± 51.7	119.2 ± 50.8	84.7 ± 15.8	0.05*
Propionate	677.3 ± 481.4	632.6 ± 581.0	442.5 ± 156.2	0.01*
Amino acids				
Alanine	92.3 ± 31.1	35.9 ± 23.2	11.3 ± 7.4	0.001***
Glycine	560.8 ± 84.9	109.3 ± 92.8	51.5 ± 35.4	0.001***
Histidine	23.3 ± 11.8	17.3 ± 4.2	^**#**^	-
Phenylalanine	82.4 ± 22.5	31.4 ± 13.8	48.5 ± 3.6	0.413
Taurine	132.4 ± 87.1	78.9 ± 32.0	48.2 ± 28.9	0.05*
Proline	182.8 ± 151.2	134.4 ± 185.0	59.1 ± 44.1	0.114
Tyrosine	115.8 ± 17.9	21.5 ± 14.4	8.6 ± 11.4	0.017*
Amines				
Trimethylamine	0.8 ± 0.6	1.9 ± 1.1	2.2 ± 1.0	0.392
Methylamine	5.7 ± 1.2	14.9 ± 1.9	20.1 ± 1.8	0.01**
Organic acids				
Citrate	12.0 ± 8.9	26.7 ± 14.4	18.3 ± 13.8	0.263
Lactate	207.5 ± 34.7	199.5 ± 27.4	178.8 ± 18.0	0.05*
Pyruvate	32.1 ± 29,0	28.7 ± 13.4	11.4 ± 4.1	0.01**
Succinate	59.9 ± 19.8	50.6 ± 15.1	47.9 ± 10.3	0.05*
Others				
Choline	21.5 ± 12.2	15.9 ± 7.2	9.5 ± 4.5	0.001***
Isopropanol	2.0 ± 1.2	2.6 ± 2.3	2.3 ± 1.3	0.394
1,2-Propanediol	69.3 ± 19.8	42.7 ± 21.1	42.7 ± 15.2	0.043*
Butanol	71.28 ± 52.7	52.3 ± 47.6	8.6 ± 5.8	0.001***
Ethanol	30.6 ± 21.6	35.5 ± 27.6	#	**-**
Fucose	694.0 ± 115.5	232.7 ± 127.8	166.3 ± 40.3	0.01***
Methanol	119.3 ± 5.1	34.1 ± 8.3	13.8 ± 4.1	0.001***

*p*-values between saliva concentrations before and after RT; *p < 0.05; **p < 0.01; ***p < 0.001 The mean difference is significant at the 0.05 level

^#^ conentrations were below the detection limit

Correlation analysis showed significant associations between different metabolites before treatment and after treatment (Fig. 1S and in Table 4). The correlation between propionate and acetate, which are the main components of propanoate metabolism, was very strong both before and after RT (0.82 and 0.97 respectively). Pyruvate exhibited strong correlations between lactate, alanine, trimethylamine, and choline. Pyruvate strongly increases its correlation with taurine and succinate after RT (0.94 and 0.87 respectively). Further, strong correlations between many metabolite pairs are presented in Table 4.

**Table 4:** The strenght of correlation between different metabolites before and after RT in HNC patients classified as follows: < 0 = no correlation; 0—0.40 = weak correlation; 0.40 – 0.60 = moderate correlation; 0.60 – 0.80 = strong correlation; > 0.80 = very strong correlation

Metabolites	Before tratment	After treatment	Fold change
Butanol – Butyric acid	**0.92**	**0.94**	0.02
Propionate – Acetate	**0.82**	**0.97**	0.15
Propionate—Proline	0.51	**0.82**	0.31
Propionate – Tyrosine	0.51	0.67	0.16
Propionate – Phenylalanine	0.14	0.65	0.51
Propionate – Formate	0.69	0.77	0.08
Propionate – Glycine	0.48	0.71	0.23
Fucose – Tyrosine	0.32	**0.82**	0.50
Alanine – Phenylalanine	0.44	**0.81**	0.37
Acetate – Proline	0.41	**0.84**	0.43
Acetate—Phenylalanine	-0.15	**0.83**	**0.98**
Acetate—Formate	0.72	**0.82**	0.10
Pyruvate – Lactate	0.06	0.69	0.53
Pyruvate – Alanine	-0.06	0.72	0.78
Pyruvate – Trimethylamine	-0.12	0.67	0.79
Pyruvate – Choline	0.03	0.78	0.75
Pyruvate – Taurine	-0.09	**0.94**	**1.03**
Pyruvate – Succinate	0.02	**0.87**	**0.85**
Succinate – Taurine	0.03	**0.92**	**0.89**
Proline – Phenylalanine	0.41	**0.86**	0.45
Proline – Formate	0.18	**0.93**	0.75
Tyrosine—Phenylalanine	-0.1	**0.97**	**0.98**
Tyrosine—Formate	0.57	0.79	0.22
Tyrosine—Glysine	0.26	**0.94**	0.68
Phenylalanine—Formate	0.15	**0.90**	0.75
Phenylalanine—Glycine	0.43	**0.89**	0.46
Formate—Glycine	0.38	**0.84**	0.46

**Fig. 2:**
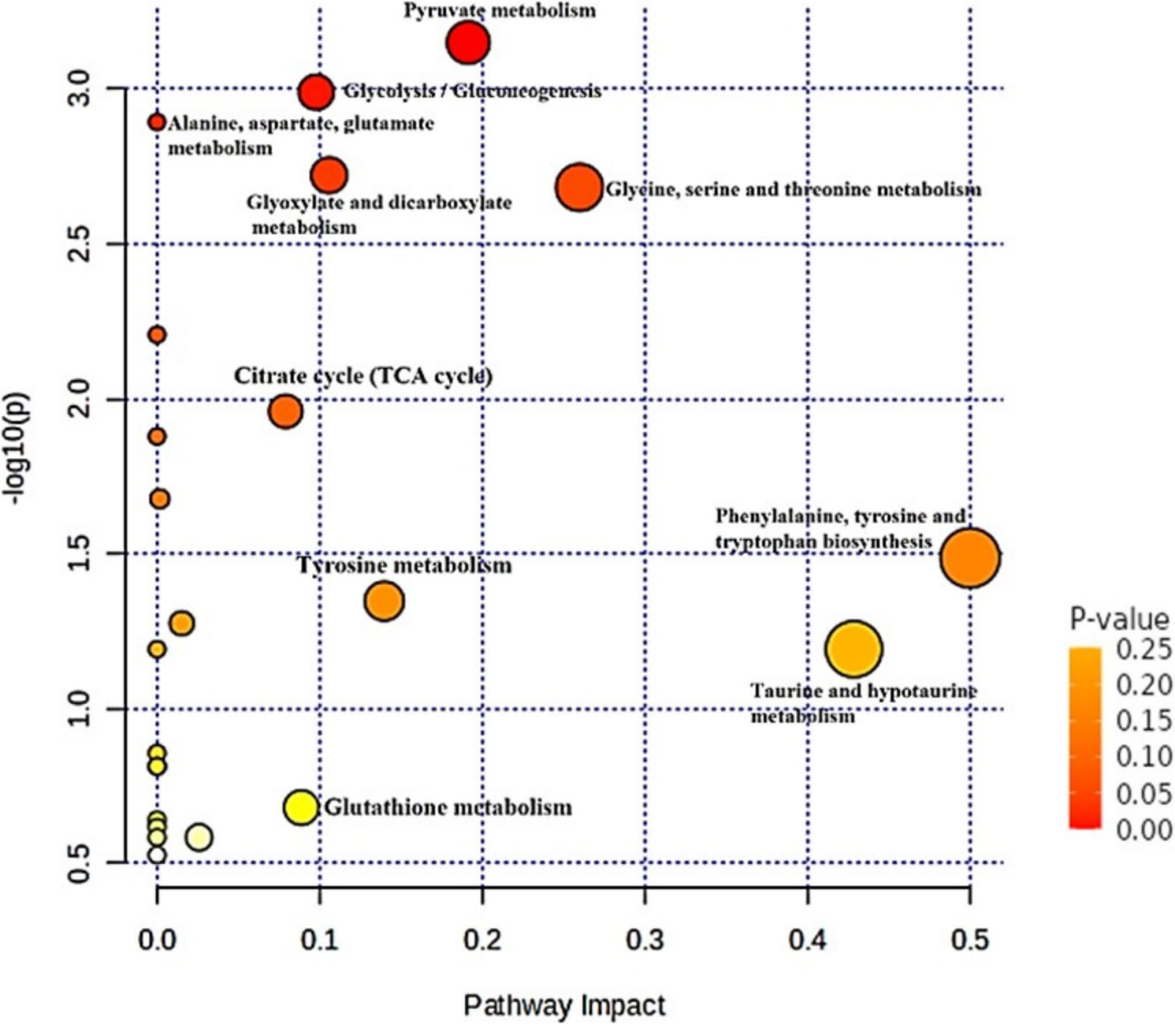
Graphical output of pathway enrichment analysis results created with MetaboAnalyst 6.0. The Y-axis represents the -log p-values from the pathway enrichment analysis, while the X-axis shows the pathway impact values. The node color and radius reflect the p-value and pathway impact value, respectively. List of pathway enrichment analysis is presented in Table 5

The altered metabolic pathways before, during and after RT were investigated by inputting the metabolites and their corresponding concentration into the open-source platform MetaboAnalyst, to make analyses in individual metabolic pathway between before and after RT study groups (Fig. 2). MetaboAnalyst considers both the number of altered metabolites in the pathway and their relative importance based on pathway topology. Pathway impact values were calculated based on the sum of the importance measures of the matched metabolites normalized by the sum of the importance measures of all metabolites in each pathway by MetaboAnalyst. The analysis revealed five pathways (Fig. 2; Table 5): 1) Pyruvate metabolism, 2) Glycolysis / Gluconeogenesis, 3) Glycine, serine and threonine metabolism, 4) Glyoxylate and dicarboxylate metabolism, and 5) Alanine, aspartate and glutamate metabolism with high significance and impact suggesting the importance of pathway during RT. The impact value and the significance (-log_10_P) of these five pathways have altered as a result of RT. These changes reflect the metabolic dysregulation in the oral cavity induced by RT. Other pathways with less impact but potential relevance include Citrate cycle-TCA cycle, Tyrosine metabolism, Phenylalanine, tyrosine and tryptophan biosynthesis, Taurine and hypotaurine metabolism, and Glutathione metabolism (Fig. 2).

**Table 5:** List of five significant pathways identified by pathway enrichment analysis: Created with MetaboAnalyst 6.0

Pathway	Total number of compounds/experimental data	Metabolites identified by NMR	-log10(p)	Patway impact
Pyruvate metabolism	20 / 4	Acetate Formate Lactate Pyruvate	3.250	0.19
Glycolysis, Gluconeogenesis	28 / 4	Acetate Ethanol Lactate Pyruvate	3.0	0.10
Glyoxylate and dicarboxylate metabolism	12 / 1	Pyruvate	2.75	0.11
Glycine, serine, and threonine metabolism	9/ 1	Pyruvate	2.70	0.26
Alanine, aspartate, and glutamate metabolism	15 / 2	Pyruvate Succinate	2.85	0

Graphical output of pathway enrichment analysis is presented in Fig. 2

## Discussion

This clinical longitudinal study was conducted to assess the potential of salivary metabolites in HNC patients detected by NMR spectroscopy. In this study, the unstimulated whole saliva from HNC patients was collected in three phases: before treatment, during treatment, and after treatment. NMR spectroscopic analysis detected certain metabolites and pathways with the potential to differentiate HNC patients from controls.

As previously shown [11, 12], salivary proline was reduced in HNC patients when compared with controls. We observed consistently decreased proline concentrations in the saliva of HNC patients during and after the treatment. The intermediates generate proline during the glycolytic and the tricarboxylic acid cycle (TCA cycle). Proline is the source of carbon exchange between the TCA cycle and the urea cycle [21]. Generally, proline is metabolized by the enzymes that respond to various stresses and contribute to redox regulation and metabolic signaling. The inter-relatedness of glutamine, glutamate, arginine, and proline has been recognized in some studies [11, 22]. It has been hypothesized that some non-essential amino acids like proline become “conditionally essential” during cancer progression. A direct, cell-autonomous role of proline synthesis or degradation in regulating the growth and survival of cancer cells is highlighted [23]. The oncogenic factor c-MYC (c-MYC), a proto-oncogene expressed in a variety of tumors including HNCs, upregulates the enzymes of proline synthesis from glutamine, so, unlike glutamine, the free amino acid pool of proline decreases [24]. In cancer, proline degradation is due to a rapid and robust increase in the transcription of proline dehydrogenase/proline oxidase by the tumor suppressor gene P53 [25]. The proline, lower concentration in HNC patients reflects the altered redox balance suggesting that proline synthesis is down regulated to conserve the NADPH/NADP^+^ balance in the mitochondrial electron transport chain, suggesting a relationship with the initiation of oral mucositis, which is related with redox alterations induced by RT and chemotherapy [26].

The concentration of salivary fucose is higher in HNC patients than in controls. Fucosylation of glycoproteins is the addition of L-fucose at the terminal end of the oligosaccharide chain. It mediates in several biological functions, and it has been found that cancer cells display abnormal fucosylation [27]. Alterations in fucosylation patterns are repeatedly associated with different malignancies [27, 28], and, in the context of HNC, elevated salivary and serum fucose levels have been reported [29, 30]. These findings are consistent with broader observations in cancer research, where increased serum fucose levels have been linked to various types of cancers [27]. The increased fucose concentration found in the saliva of HNC patients, compared to controls, is likely due to abnormal fucosylation, and it was reported that tumour cell fucosylation changes influence cell-to-cell recognition, cell adhesion, metastasis, and tumour growth [27, 30].

Our results showed the increased fucose levels in our HNC patients compared with controls indicating that the altered tissue or the cancerous tissue has released fucose into the saliva, probably due to increasing the turnover and release of cells. Also, it is emphasized that the cancer cells overexpress fucosylated glycans on their surface and are continuously shed into the saliva. It is suggested that the increased presence of fucose is also caused by more local synthesis by cancer cells than the destruction of the malignant cells [27]. In the present study, RT reduces the concentration of fucose in saliva; this highlights the potential of fucosylation as a diagnostic and prognostic tool in cancer treatment, and as a potential metabolic biomarker of treatment response. Though fucose plays a role in HNC its changes in cancer are understudied, future research is necessary.

It is reported that 1,2-propanediol (PPD) acts as a radioprotector against radiation-induced hematopoietic injury [31]. A reduction of 1,2-propanediol was observed during and after the treatment in the saliva of our HNC patients. Such a reduction, after the radiation therapy in HNC patients, might indicate the appearance of radiation-induced oral toxicities and other side effects related to RT.

Pathway analysis revealed alterations in several key metabolic pathways. Pyruvate participates in multiple biosynthetic pathways, and it maintains cellular NAD + levels to ensure the continuation of glycolysis and ATP production. It was reported that an aberrant pyruvate metabolism plays an prominent role in cancer, and that cancer cells modulate pyruvate metabolism by the downregulation of p53 and by shifting the expression of glycolytic enzymes, such as pyruvate kinase [32]. The Pasteur effect, in anaerobic glycolysis, is established as a cellular adaptation to hypoxia and the Warburg effect, in aerobic glycolysis, is a part of the metabolic transformation in cancer cells. In both situations, pyruvate supports the ATP production and other NAD + -dependent metabolism [33]. Cancer cells utilize more glucose that upregulates gluconeogenic pathways. Lactate and alanine are potential glucose precursors for gluconeogenesis [34]. It was reported that an aberrant pyruvate metabolism plays a prominent role in cancer, and that cancer cells modulate pyruvate metabolism by the downregulation of p53 and by shifting the expression of glycolytic enzymes, such as pyruvate kinase [31]. Similarly, alanine, aspartate and glutamate metabolism pathways are reported to be associated with mutant p53 status [35]. Also, altered glutamine metabolism and TCA cycle are associated with several types of cancer [36, 37]. Glutamine metabolism is important in cells under pathophysiological conditions. It contributes its carbon backbone to the TCA cycle anaplerosis in cancer cells and activates glycolysis and glutamine-dependent TCA cycle. Glutamine is required for enhanced hexosamine biosynthesis pathway flux in cancer cells [38]. In addition, glutamine-derived glutathione plays a key role in tightly controlling reactive oxygen species metabolism to maintain redox balance [39]. Choline provides membrane stability and has functions in osmoregulation and cell signaling. The abnormal choline metabolism before RT hints at cell proliferation dysregulation [40, 41] compared to controls and the subsequent decrease in choline during and after RT might indicate a break in cell proliferation. The increase and decrease in choline was observed in several studies suggesting alteration in phospholipid metabolism, lipid, lysine and fatty acid metabolism [42–44].

In the present study, 3 metabolites, proline, fucose and 1,2-propandiol were identified as discriminatory salivary metabolites in HNC patients relative to healthy subjects. However, the levels of SCFAs (acetate, formate, propionate), glycine, tyrosine, methylamine, lactate, pyruvate, choline, butanol and methanol were also slightly increased in HNC patients, probably due to the different metabolic rates (for maintaining a homeostatic state) in the different biological compartments (serum and blood). Such alterations could be in response to pathophysiological stimuli. The salivary metabolites observed in HNC patients before, during and after the treatment presented a consistent decrease in 17 metabolites (Table 3). Hence, it can be speculated that RT in HNC patients has some effect on the salivary metabolic pathways. Although, it is difficult to comment on its exact mechanism. But the effect of RT on salivary gland dysfunction, decreased saliva, increased inflammation and oral dysbiosis are previously described [45].

The adverse effect of RT treatment and patient outcomes among patients with HNC highlights the need for biomarkers that predict their severity and prognosis. However, several risk factors including the type of chemotherapy regimen, radiation dose, inadequate oral hygiene, alcohol, and history of smoking, have been established that affect the treatment outcome [46]. Saliva is collected non-invasively which gives insight into patients’ status. We observed a decrease in the concentration of most of the salivary metabolites after RT. A few of the metabolites i.e., amines, citrate, and isopropanol concentrations were slightly increased after treatment, only methylamine was increased significantly. A rise in certain salivary metabolite levels during RT suggests a decreased protective effect of the oral mucosa, potentially due to a decrease in saliva secretion, and high susceptibility to infection [47]. Future studies may ascertain the origin of these metabolites (mammalian cells, oral microbiome or combination) and shed light on the potential pathways associated with the development or progression of oral health during cancer treatment, for example, the prognosis of oral mucositis with salivary metabolomics.

Studies in salivary metabolomic are mainly focused on the investigations of specific biomarkers in different diseases. However, understanding the pathological pathways in the oral cavity is of great importance for mapping the molecular attributes of oral diseases. In the future, studies on the interaction between salivary pathways and oral microbiota may generate data of interest in the whole body, such as gut, skin, brain, and lung.

## Conclusion

This study demonstrates that salivary metabolomics provide valuable insights into the information about RT-induced pathobiological changes in HNC patients. Five salivary pathways have shown to be changed during RT. These changes in the oral biological system may reflect underlying processes, such as redox homeostasis, oxidative stress and inflammation. The ability to monitor salivary pathways non-invasively holds promise to personalized medicine in HNC treatment. While this study provides valuable insights into the salivary metabolic pathways of HNC patients undergoing RT, further research with larger cohorts and longer follow-up periods is needed to validate these findings and establish their clinical utility.

## Supplementary Information

Below is the link to the electronic supplementary material.

ESM 1 (JPG 204 KB)

## Data Availability

No datasets were generated or analysed during the current study.
