# Correction to: Radiation-induced changes in salivary metabolite profile and pathways in head and neck cancer patients

**DOI:** 10.1007/s00784-025-06251-2

**Published:** 2025-03-10

**Authors:** Saga Ramsay, Eelis Hyvärinen, Wilfredo González-Arriagada, Tuula Salo, Marcio Ajudarte Lopes, Jopi J. W. Mikkonen, Bina Kashyap, Arja M. Kullaa

**Affiliations:** 1 Institute of Dentistry, School of Medicine, University of Eastern Finland, Kuopio Campus, 70210 Kuopio, Finland; 2 Educational Dental Clinic, Kuopio University Hospital, The Wellbeing Services County of North Savo, Kuopio, Finland; 3 Facultad de Odontología, Universidad de los Andes, Santiago, Chile; 4 Centro de Investigación E Innovación Biomédica, Universidad de los Andes, Santiago, Chile; 5 IMPACT-Center of Interventional Medicine for Precision and Advanced Cellular Therapy, Santiago, Chile; 6 Department of Pathology, University of Helsinki and Helsinki University Hospital, Helsinki, Finland; 7 Department of Oral and Maxillofacial Diseases, Faculty of Medicine, University of Helsinki, Helsinki University Hospital, ClinicumHelsinki, Finland; 8 Translational Immunology Research Program (TRIMM), University of Helsinki, Helsinki, Finland; 9 CAN Digital Precision Cancer Medicine Flagship, University of Helsinki, Helsinki, Finland; 10 Research Unit of Population Health, Faculty of Medicine, University of Oulu, Oulu, Finland; 11 Medical Research Center, Oulu University Hospital, University of Oulu, Oulu, Finland; 12 Department of Oral Diagnosis, School of Dentistry, State University of Campinas, Sao Paulo, CEP 13414-018 Brazil

**Correction to: Clinical Oral Investigations**

10.1007/s00784-025-06225-4

In Figure 1 caption (Some of the text in Figure 1 has gone to page 7 as text in Figure 2):

**Fig. 1 **Workflow of saliva collection in HNC patients at three different time points; 1^st^ one week before radiotherapy (RT), 2^nd^ during RT, and 3^rd^ one week after RT

Figure 2 caption should be:

**Fig. 2** Graphical output of pathway enrichment analysis results created with MetaboAnalyst 6.0. The Y-axis represents the -log p-values from the pathway enrichment analysis, while the X-axis shows the pathway impact values. The node color and radius reflect the *p*-value and pathway impact value, respectively. List of pathway enrichment analysis is presented in Table 5

The original article has been corrected.

