# Supplementary figures and images for: Radiation-induced changes in salivary metabolite profile and pathways in head and neck cancer patients
Source: Clin Oral Investig. 2025 Feb 21;29(3):145. doi: 10.1007/s00784-025-06225-4 (PMC11845554; doi:10.1007/s00784-025-06225-4)

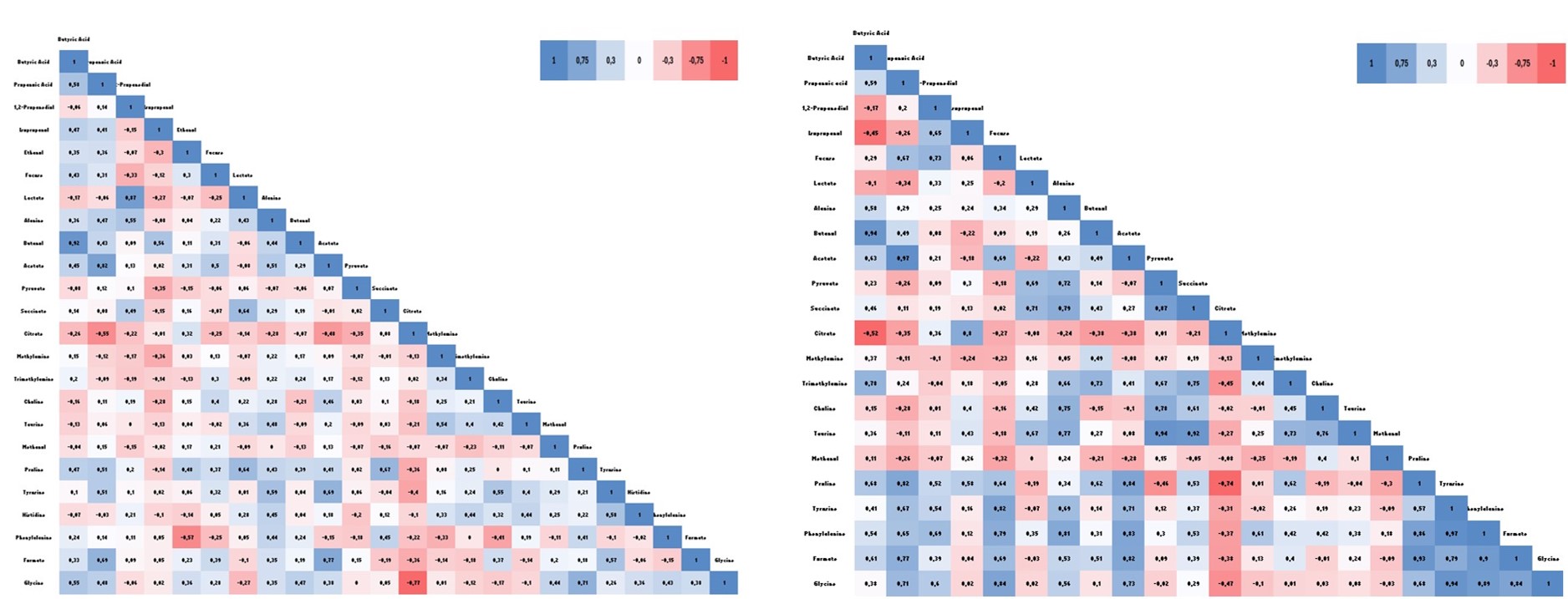

Supplement: Supplementary file 1 — (JPG 204 KB) [file 784_2025_6225_MOESM1_ESM.jpg]
